# High Sensitivity of *Giardia duodenalis* to Tetrahydrolipstatin (Orlistat) *In Vitro*

**DOI:** 10.1371/journal.pone.0071597

**Published:** 2013-08-19

**Authors:** Juliane Hahn, Frank Seeber, Herbert Kolodziej, Ralf Ignatius, Michael Laue, Toni Aebischer, Christian Klotz

**Affiliations:** 1 Institute of Pharmacy, Pharmaceutical Biology, Freie Universität Berlin, Berlin, Germany; 2 Mycotic and Parasitic Agents and Mycobacteria, Department of Infectious Diseases, Robert Koch-Institut, Berlin, Germany; 3 Institute of Tropical Medicine and International Health, Charité – Universitätsmedizin Berlin, Berlin, Germany; 4 Advanced Light and Electron Microscopy, Centre for Biological Threats and Special Pathogens, Robert Koch-Institut, Berlin, Germany; National University of Singapore, Singapore

## Abstract

Giardiasis, a gastrointestinal disease caused by *Giardia duodenalis*, is currently treated mainly with nitroimidazoles, primarily metronidazole (MTZ). Treatment failure rates of up to 20 percent reflect the compelling need for alternative treatment options. Here, we investigated whether orlistat, a drug approved to treat obesity, represents a potential therapeutic agent against giardiasis. We compared the growth inhibitory effects of orlistat and MTZ on a long-term *in vitro* culture adapted *G. duodenalis* strain, WB-C6, and on a new isolate, 14-03/F7, from a patient refractory to MTZ treatment using a resazurin assay. The giardiacidal concentration of the drugs and their combined *in vitro* efficacy was determined by median-effect analysis. Morphological changes after treatment were analysed by light and electron microscopy. Orlistat inhibited the *in vitro* growth of *G. duodenalis* at low micromolar concentrations, with isolate 14-03/F7 (IC50_24h_ = 2.8 µM) being more sensitive than WB-C6 (IC50_24h_ = 6.2 µM). The effect was significantly more potent compared to MTZ (IC50_24h_ = 4.3 µM and 11.0 µM, respectively) and led to specific undulated morphological alterations on the parasite surface. The giardiacidal concentration of orlistat was >14 µM for 14-03/F7 and >43 µM for WB-C6, respectively. Importantly, the combination of both drugs revealed no interaction on their inhibitory effects. We demonstrate that orlistat is a potent inhibitor of *G. duodenalis* growth *in vitro* and kills parasites at concentrations achievable in the gut by approved treatment regimens for obesity. We therefore propose to investigate orlistat in controlled clinical studies as a new drug in giardiasis.

## Introduction

Giardiasis is caused by the protozoan parasite *Giardia duodenalis* (syn. *G. intestinalis, G. lamblia*) and is one of the most common parasitic diseases worldwide [1]. It has a significant impact on public health in both developing and developed countries and was included in the ‘neglected disease initiative’ of the WHO in 2004 [2]. The life cycle of *Giardia spp*. is direct and includes flagellated trophozoites and non-proliferative, infectious cysts that are transmitted via fecal-oral routes. After ingestion parasites hatch and undergo transformation into rapidly multiplying trophozoites that attach to the epithelial layer of the upper small intestine.

*G. duodenalis* infections remain either asymptomatic or induce severe and/or chronic (relapsing) disease symptoms, and therefore treatment is generally indicated [3]. The mechanisms underlying the pathogenesis of giardiasis are not well understood but presumably depend on both host and parasite factors [1]. For example, the *G. duodenalis* species complex consists of eight main genotype groups (assemblages) that are morphologically identical but differ in host specificity. Assemblages A and B show the broadest host specificity and are the only assemblages that are pathogenic in humans. Recent data have implied strain-specific pathogenicity of different genotypes and possibly sub-genotypes in rodent models [4], [5]. In humans, however, association between specific genotypes and clinical symptoms have been inconclusive so far [6].

Metronidazole (MTZ) is the first choice for the treatment of giardiasis, with other nitroimidazoles (e.g. tinidazole) as alternatives. The current model of the mode of action of MTZ proposes its intracellular reduction to a toxic radical form by various parasite reductases, including pyruvate:ferredoxin oxidoreductase, nitroreductase and thioredoxin reductase [7]–[9]. Alternative compounds include albendazole, nitazoxanide, paromomycin and furazolidone. However, most of the therapeutically used antigiardial drugs, including MTZ cause severe side effects and are not well tolerated by many patients [3]. Furthermore, clinical resistance to medication has been observed for all common drugs in up to 20% of giardiasis cases [3], [10], [11]. Treatment failure may be due to both host factors (e.g. low patient compliance due to side effects) and parasite resistance. The latter has been shown by several studies demonstrating marked differences in the *in vitro* drug susceptibility of isolates from patients [4], [12]–[14]. The limitations of current antigiardial drugs emphasize the requirement of new, efficient and well-tolerated therapeutics [10], [11], [15]. To this end, reprofiling of compounds that have been approved for the use in humans is a valid strategy [16]–[18].

An efficient lipid metabolism is a sine qua non condition for rapid proliferation and survival of living organisms. Current data suggest that *G. duodenalis* parasites possess only restricted resources to synthetize and to metabolize lipids [19]. These parasites thus depend strongly on the exploitation of lipids supplied by the host environment, rendering therapeutic targeting of enzymes associated with their lipid metabolism a promising strategy [20].

Tetrahydrolipstatin (orlistat), a derivative of the naturally occurring lipase inhibitor lipstatin from *Streptomyces toxytricini*, has been approved for the treatment of obesity due to its potent inhibition of pancreatic lipases [21], [22]. It is widely used in humans for this purpose and thus can be considered relatively safe [23]–[26]. Recently, orlistat has been shown to inhibit the growth of pathogens, including apicomplexan parasites and trypanosomes, and that of cancer cells *in vitro* [18], [27]–[29]. These pharmacological activities indicate that orlistat has anti-microbial properties and effects on host cells that may be therapeutically exploitable.

The aim of the present study was to evaluate orlistat for its *in vitro* growth inhibitory potential on *G. duodenalis* alone and in comparison with MTZ. Our data show a more potent effect of orlistat on *G. duodenalis* replication *in vitro* and suggest that the combination of both drugs may be an appropriate treatment option for giardiasis.

## Materials and Methods

### G. duodenalis Strains

The WB-C6 strain of *G. duodenalis* was derived from the American Type Culture Collection (ATCC #50803; genotype AI). Isolate 14-03/F7 was obtained from a human patient chronically infected and refractory to treatment with MTZ by *in vitro* excystation following the protocol of Rice and Schaefer [30]. Briefly, cysts were enriched from a fresh stool sample by 1 M sucrose gradient flotation. For *in vitro* excystation 250 µL (ca. 2.5×10^5^ cysts) water-resistant cysts were pre-incubated with 250 µL of an antibiotics mixture (final concentration: erythromycin 136 µM, chloramphenicol 613 µM, amikacin 342 µM, tetracycline 450 µM, rifampicin 243 µM) for 30 min, followed by addition of 10 mL acidic excystation solution I (5 mL HCl pH = 2.0; 2.5 mL Hank’s buffered salt solution containing 29 mM L-cysteine HCl and 67 mM glutathione; 2.5 mL 0.1 M sodium bicarbonate). After incubating at 37°C for 30 min cysts were pelleted at 900×*g* for 5 min, washed once in 10 mL excystation solution II (0.5% trypsin dissolved in Tyrode solution) and incubated in 1 mL excystation solution II for an additional 30 min at 37°C. Finally, cysts were sedimented and resuspended in modified TYI-S-33 culture medium containing an antibiotics cocktail of lower final concentrations (erythromycin 2.7 µM, chloramphenicol 12.3 µM, amikacin 6.8 µM, tetracycline 9.0 µM, rifampicin 4.9 µM, fosfomycin 724 µM, penicillin 100 U/mL, streptomycin 172 µM). Serial dilutions of excysting parasites were seeded into a 96-well plate (200 µL per well) and incubated under oxygen-deprived conditions (see below) at 37°C.

The isolate 14-03/F7 was derived from one well and was genotypically characterized at the triosephosphate isomerase locus by PCR-product sequencing according to a published protocol [31]. Sequence analysis using defined reference sequences [32] revealed 100% homology to the reference sequence of AII (Genbank accession no. U57897).

### Growth Conditions

*G. duodenalis* trophozoites were cultured axenically in 11 mL screw-cap culture tubes (Nunc) at 37°C in filter-sterilised modified TYI-S-33 medium containing 10% fetal bovine serum as previously described [33]. Tubes were filled with at least 10 mL culture medium to ascertain low-oxygen conditions. Cultures were split two to three times a week by placing confluent cultures on ice for 30 min to facilitate detachment of trophozoites that were further cultured after dilution (1∶100 or 1∶1000) with fresh culture medium as described above.

To ascertain optimal oxygen-restricted growth conditions for *G. duodenalis* trophozoites that were cultured in cell culture plates or Petri dishes, the parasites were incubated at 37°C for indicated times in Aerogen Oxoid jars containing appropriate reaction bags (Oxoid #AN0025).

### Resazurin-assay

Drug susceptibility of *G. duodenalis* parasites was determined using a previously described resazurin assay [34] with some modifications. Briefly, trophozoites were harvested by incubating the cultures on ice for at least 30 min, followed by centrifugation at 900×*g* for 10 min at 4°C. Trophozoite pellets were resuspended in modified TYI-S-33 medium, counted using a Neubauer chamber and seeded in 200 µL/well of modified TYI-S-33 medium into black polystyrene 96-well plates with flat clear bottom (Costar Corning). Trophozoites were incubated at 37°C under oxygen-restricted growth conditions (see above). To optimize assay conditions, parasite numbers of 0.5×10^3^–1×10^4^ per well were tested. These assays revealed an adequate inoculum size of 5×10^3^ trophozoites per well that was used for all subsequent experiments (see results). After 24 h trophozoites were washed with 200 µL warm (37°C) phosphate-buffered-saline (PBS), pH 7 and incubated with 220 µL of resazurin (Sigma R7017, analytical grade) in PBS per well (final concentration 20 µM) for 4 h at 37°C under oxygen-deprived conditions. The redox-sensitive dye resazurin changes from blue/non-fluorescent to the pink/highly-fluorescent resorufin when reduced by metabolically active trophozoites. Fluorescence intensity was measured fluorometrically at 595 nm using a 550 nm excitation wavelength (Tecan Infinite M200 Pro).

To test drug susceptibility trophozoites were incubated with serial dilutions of the compounds individually or in combination following the standardised protocol as described above. Stock solutions of MTZ (Sigma 46461, analytical standard) and of orlistat (Sigma O4139, purity >98%) were prepared in DMSO and ethanol, respectively. The final volume of diluent did not exceed 0.12% (v/v) and had no significant effect on growth of both *G. duodenalis* isolates (data not shown). For data analysis, the fluorescence intensities of treated trophozoites were correlated with the values of non-treated trophozoites as a control group set to 100% (percentage of growth). The half maximal inhibitory concentrations (IC50_24h_) were calculated from viability plots of serial drug dilutions using Prism 5 software (GraphPad). The subscript index indicates the respective assay time used to calculate the IC50 value (e.g., IC50_24h_ = 24 h assay time)_._

### Calculation of Replication Times

The replication times of the *G. duodenalis* isolates were derived from cultures (in triplicates) with an inoculum of 1×10^5^ parasites in 10 mL modified TYI-S-33 medium cultured under oxygen-restricted growth conditions (see above). After 24, 48, 72, and 96 h, numbers of trophozoites were counted using a Neubauer counting chamber. For this, the cultures were incubated on ice for at least 30 min and the parasites collected by centrifugation at 4°C and 900×*g*. The doubling time during the log phase was calculated from growth curves using Prism 5 software.

### Cell Staining and Light Microscopy

Trophozoites were seeded onto round cover slips placed into a 24-well plate and incubated in the absence or presence of orlistat (0.1–37.5 µM) in modified TYI-S-33 medium. After 24 h, Romanowsky staining of the trophozoites was performed according to the manufacturer’s protocol (Diff-Quik®, Medion Diagnostics). Cover slips were mounted in Entellan mounting medium (Merck) and trophozoite morphology was imaged using an Axio Observer microscope (Carl Zeiss Microscopy).

### Electron Microscopy

Cultures of treated and untreated *G. duodenalis* trophozoites (WB-C6 strain) were prepared for scanning (SEM) and transmission (TEM) electron microscopy to reveal the overall morphology of the trophozoites and ultrastructural elements. Trophozoites were grown on glass coverslips (1×10^5^ trophozoites, for SEM) or in plastic petri dishes (1×10^7^ trophozoites, for TEM) for 24 h at 37°C under oxygen-restricted conditions (see above).

Processing for SEM: Culture medium was removed and adherent trophozoites were briefly washed with PBS at 37°C before the pre-warmed fixative, consisting of 4% paraformaldehyde, 2.5% glutaraldehyde in 0.1 M phosphate buffer (pH 7.2), was added. Coverslips with adherent trophozoites were post-fixed with osmium tetroxide (1% in distilled water, for 1 h), dehydrated in a graded series of ethanol and dried by critical point drying using carbon dioxide (Emitech K850; Quorum Technologies). Then, coverslips were mounted onto sample stubs and coated with a thin layer (about 3 nm) of gold-palladium (Sputter Coating Unit E5100; Polaron). Finally, samples were examined with a LEO 1530 field-emission scanning electron microscope (Carl Zeiss Microscopy) at 5 kV using the in-lens secondary electron detector.

Processing for TEM: For fixation, culture medium was removed and adherent trophozoites were briefly washed with PBS at 37°C, before the pre-warmed fixative, consisting of 4% paraformaldehyde, 2.5% glutaraldehyde in 0.1 M phosphate buffer (pH 7.2), was added. After 2 h incubation at RT trophozoites were stored at 4°C overnight. In parallel, the supernatant collected from the petri dishes was cooled to 4°C and centrifuged at 900×*g* for 10 min to harvest the trophozoites which had detached from the culture dish surface. The pellet was washed with PBS at 4°C, again centrifuged at 900×*g* for 10 min and resuspended with pre-cooled fixative (composition as above) and fixed overnight at 4°C. The trophozoites were mixed with fixed trophozoites that had been scraped from the surface of the corresponding petri dish. The trophozoites were combined in order to analyse all respective trophozoites of a given treatment in a single sample which takes into account that the number of detached cells was too low for an analysis in some of the treatments. The pooled trophozoites were embedded in low-melting point agarose and subsequent processing steps included post fixation in osmium tetroxide (1% in distilled water), block contrasting with tannic acid (0.1% in 0,05 M Hepes) and uranyl acetate (2% in distilled water), dehydration and embedding in epon resin as described by Laue [35]. Ultrathin sections were prepared using an ultramicrotome (UC7; Leica) and stained with uranyl acetate (2% in distilled water, 20 min) and lead citrate (3 min) to increase contrast. The sections were examined using a Tecnai12 transmission electron microscope (FEI) operated at 120 kV.

### Drug Combination Analysis

The combined effect of MTZ and orlistat on *G. duodenalis* growth was determined using the median-drug effect analysis that involves (i) generation of median-effect plot; (ii) calculation of drug doses for a given degree of effect; and (iii) calculation of the combination index (CI) as described by Chou and Talalay [36]. To this end, *G. duodenalis* trophozoites were incubated with MTZ (0.33–81 µM) or orlistat (0.33–27 µM) individually and in combination at different molar ratios of MTZ to orlistat (1∶1, 3∶1, 1∶3 respectively). The CIs for a given degree of inhibition (fa = fraction affected) were calculated and graphically plotted to determine whether the underlying combination effect was synergistic, antagonistic, or not interactive. The interaction of the combination was defined as showing ‘synergy’ if the CI was <0.8, ‘no interaction’ effects if the CI was in the range 0.8–1.2, and ‘antagonism’ if the CI was >1.2 [37], [38].

### Test of Giardiacidal versus Giardiastatic Effects

Trophozoites were seeded in 24-well plates at a density of 5×10^4^ parasites/well (WB-C6) or of 1×10^5^ parasites/well (14-03/F7) and subsequently incubated with various drug concentrations as indicated for 24 h at 37°C. Then the trophozoites were detached by incubation on ice for 30 min. All trophozoites including non-adherent parasites were harvested, pelleted by centrifugation for 5 min at 900×*g* and 4°C and finally washed three times with 5 mL ice-cold PBS to completely remove the drug. An aliquot was taken to calculate total trophozoite numbers after drug treatment. The remaining trophozoites were transferred to 11 mL culture tubes and incubated for 48 h at 37°C in complete modified TYI-S-33 medium as described above to determine whether the drugs had effectively killed the trophozoites or whether trophozoites resumed growth. Total trophozoite numbers were determined after 24 h and 48 h, respectively. The detection limit of the counting assay was 2.75×10^3^ total trophozoites.

### Statistical Analysis

Statistical analyses were performed using the software program Prism 5.01 (GraphPad) as stated in the figure legends. Accepted significance level was p<0.05.

## Results

### Potent Inhibition of G. duodenalis Replication by Orlistat

**Figure 1:**
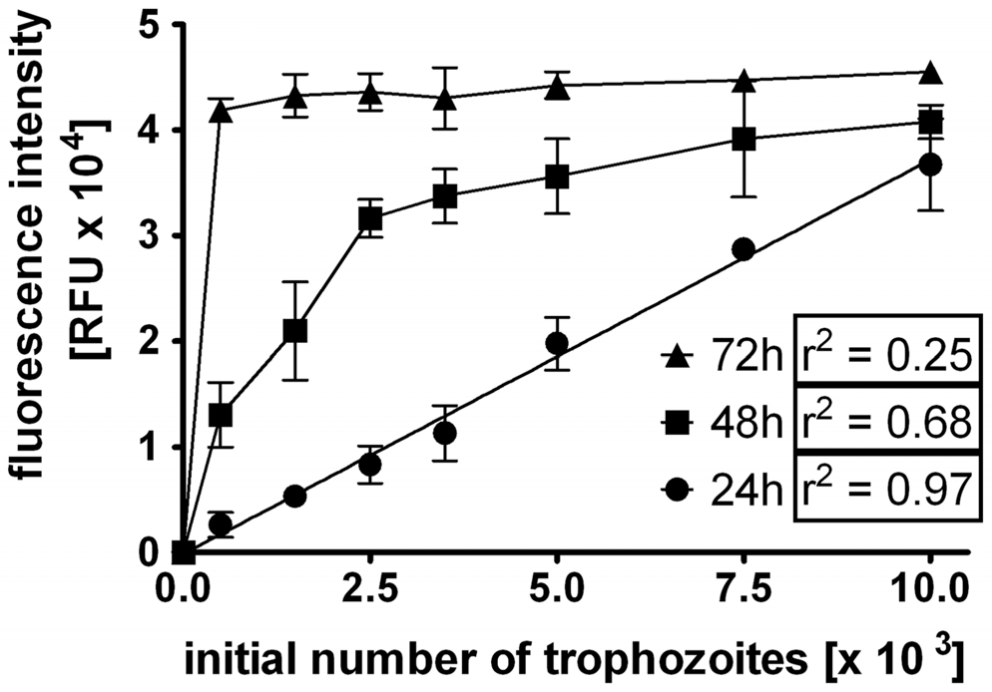
Establishment of a resazurin-based *G. duodenalis* growth assay. Various numbers of trophozoites (WB-C6 strain, 0.5×10^3^–10^4^ trophozoites per well) were seeded into 96-well plates. After 24 h, 48 h or 72 h of culture, the medium was replaced by PBS and resazurin solution was added. Trophozoites were further incubated for 4 h and the fluorescence intensity was measured fluorometrically using an excitation wavelength of 550 nm and an emission wavelength of 595 nm. Results are presented as fluorescence intensity (RFU) in relation to the initially inoculated number of trophozoites; Data are expressed as mean ± SD of three independent experiments. The correlation factor (r^2^) was calculated by linear regression using the software program Prism 5.01 (GraphPad).

To determine the *in vitro* drug susceptibility of *G. duodenalis* WB-C6 trophozoites, we first established a previously described resazurin to resorufin conversion assay [34]. However, testing inoculum sizes from 0.5×10^3^ to 1×10^4^ trophozoites and incubation times of 24 h–72 h indicated a non-linearity between parasite numbers and fluorescence intensity of resorufin at 48 h and 72 h (Fig. 1). Moreover, adopting an inoculum size of 5×10^4^ parasites and an incubation time of 72 h in a 96-well plate as originally described [34] produced in our hands data that were out of the linear range (data not shown). This is most likely due to over-growing effects or insufficient surface for attachment of proliferating parasites, resulting in loss of parasites during washings. By contrast, satisfactory data sets were achieved with inoculum concentrations between 0.5×10^3^ and 10^4^ parasites/well and an incubation time of 24 h followed by cultivation with resazurin for another 4 h. Under these conditions a linear correlation between parasite numbers and fluorescence intensity was determined (Fig. 1). For subsequent analysis we therefore used standardised conditions of 5×10^3^ parasites/well and 24 h incubation time (see methods for details).

**Figure 2:**
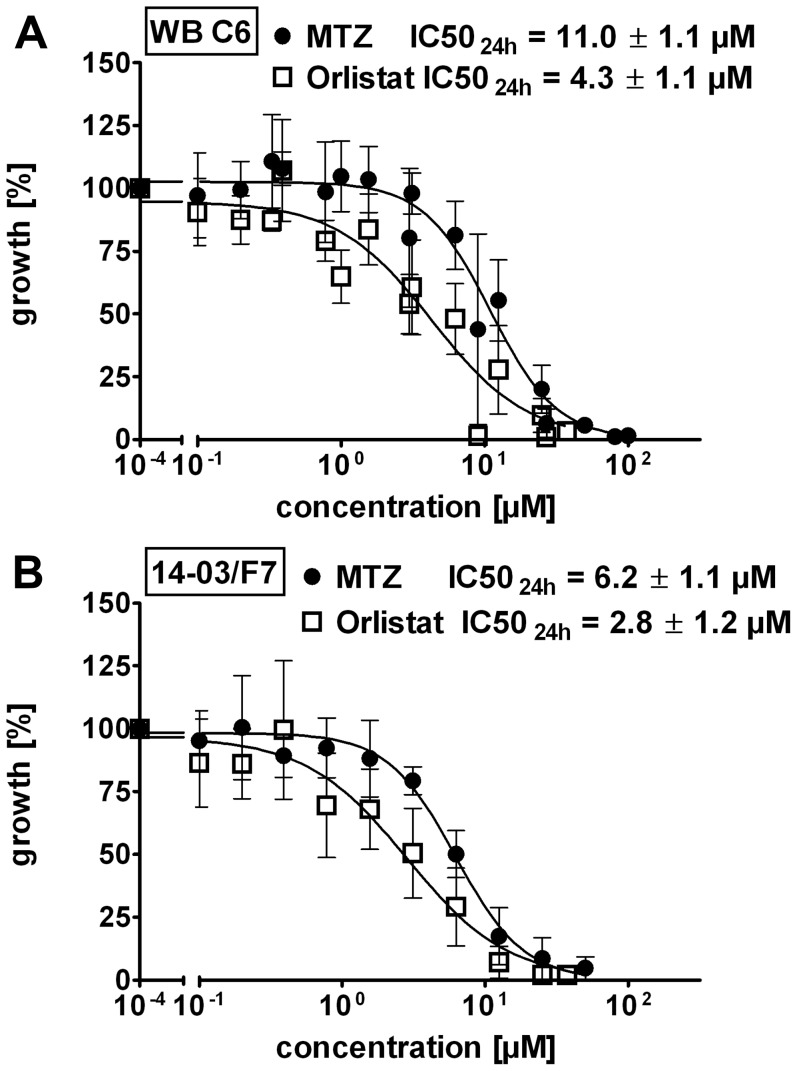
Orlistat inhibits growth of *G. duodenalis* trophozoites. ****** Anti-*G. duodenalis* activity of MTZ (filled circle) and orlistat (blank squares) was determined in a resazurin assay and results are presented as relative percentage of growth of the untreated trophozoites as a control. In addition, the half maximal inhibitory concentrations (IC50_24h_) are shown. (A) Growth inhibition curve of the laboratory strain WB-C6; the IC50_24h_ of orlistat (IC50_24h_ = 4.3 µM; n = 7) was significantly lower than the IC50_24h_ of MTZ (IC50_24h_ = 11.0 µM; n = 10), p = 0.0034. (B) Growth inhibition curve of the *G. duodenalis* patient isolate 14-03/F7; the IC50_24h_ of orlistat (IC50_24h_ = of 2.8 µM, n = 4) was significantly lower than the IC50 of MTZ (IC50_24h_ = 6.2 µM; n = 5), p = 0.0079. Data are expressed as mean ± SD. The Mann-Whitney-test was used to evaluate the statistical significance of the differences in IC50 values.

Treatment of WB-C6 trophozoites with orlistat revealed a potent and dose-dependent inhibition of parasite replication compared to the control (Fig. 2a). The IC50_24h_ of orlistat (4.3±1.1 µM) was significantly (p = 0.0039) lower than that of MTZ (11.0±1.1 µM). Next, we tested orlistat for its growth inhibitory effects on a newly established *G. duodenalis* isolate (14-03/F7) derived from a patient with chronic giardiasis who was refractory to MTZ treatment. Orlistat (IC50_24h_ of 2.8±1.2 µM) was again significantly (p = 0.0079) more effective when compared with MTZ (IC50_24h_ of 6.2±1.1 µM) (Fig. 2b). The observed higher sensitivity of the freshly established isolate to the drugs may in part be related to slower *in vitro* growth characteristics [4]. Isolate 14-03/F7 has an apparent generation time of 14 h which is nearly twice the time of 8 h required by the WB-C6 strain.

### Orlistat Treatment Leads to Altered Morphology and Detachment of *G. duodenalis* Trophozoites *in vitro*

**Figure 3:**
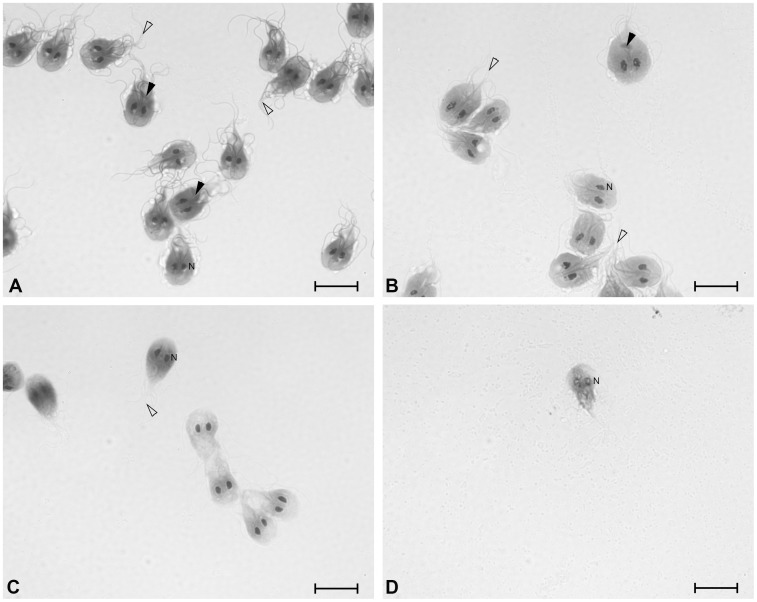
Orlistat treatment leads to altered morphology of *G. duodenalis* trophozoites. To analyse trophozoite morphology WB-C6 trophozoites were incubated with increasing orlistat concentrations for 24 h and stained with a Romanowsky stain (DiffQuick). Median body (black triangle); Flagella (triangle); Nucleus (N). A control, B 3.1 µM, C 6.3 µM and D 25 µM orlistat. Scale bar = 10µm.

Microscopic inspection of trophozoites treated with increasing concentrations of orlistat revealed a degenerated morphology. Many parasites detached from the culture plate and were less motile. To analyse the morphology in more detail, we performed Romanowsky staining of untreated and orlistat-treated trophozoites. Untreated trophozoites showed the typically pear-shaped body, pink/purple staining of the cytoplasm and flagella, and two dark purple nuclei. Upon increasing orlistat concentration, the flagella of the trophozoites that were still attached to the glass cover slip became only faintly stained and showed signs of structural degeneration. The median body was no longer detected and the parasites lost their clear pear shape of the cell body (Fig. 3).

**Figure 4:**
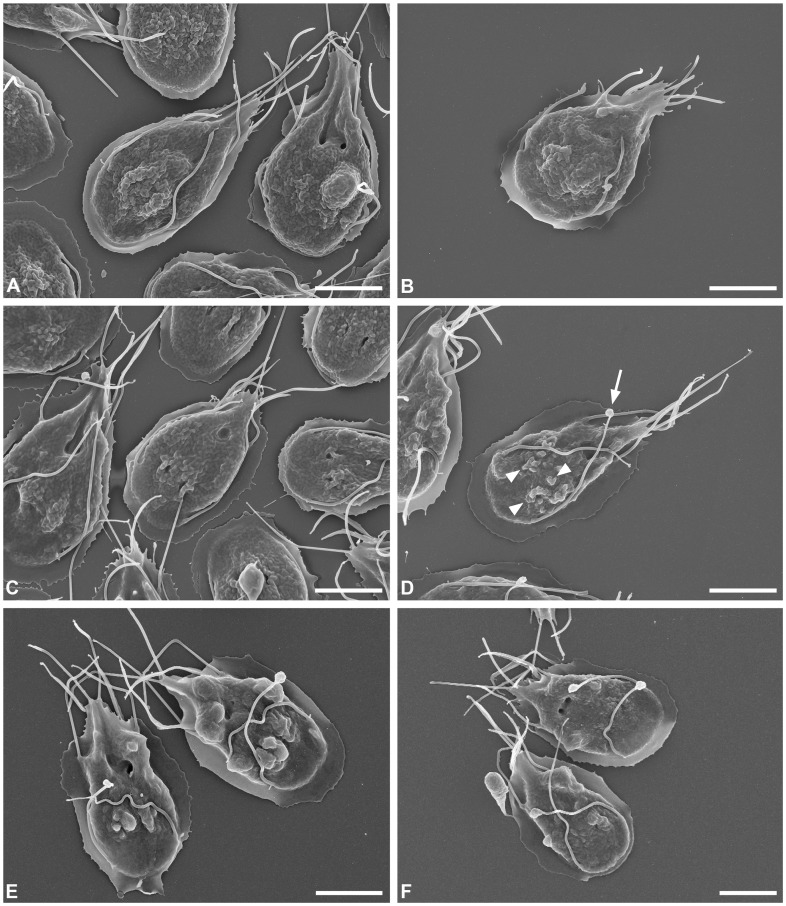
Scanning electron microscopy of orlistat-treated *Giardia duodenalis* trophozoites (WB-C6 strain) showing altered morphology. (A) Solvent control (DMSO, 0.1% in medium). (B) MTZ, 11 µM (C) Solvent control (ethanol, 0.12% in medium). (D, E, F) Orlistat, 5 µM. Controls (A, C) reveal the typical appearance of trophozoites with their pear-shaped cell body and the characteristic arrangement of the eight flagella. MTZ treated trophozoites (B) possess shorter flagella than the corresponding control trophozoites (A). Orlistat treated trophozoites (D, E, F) appear shrunken in comparison to corresponding control trophozoites (C) and show blebs on their dorsal surface (arrowheads) as well as at the tip of the flagella (arrow) (D). (E, F) In some of the trophozoites orlistat mediated effects (blebs at flagella and dorsal cell surface) are more prominent. Scale bar = 5 µm.

To further investigate morphological changes *G. duodenalis* trophozoites (WB-C6 strain) were analysed by electron microscopy to reveal ultrastructural changes due to treatment with either orlistat or MTZ in comparison to respective controls (untreated and solvent treated trophozoites). Treatment of adhering trophozoites with the drugs induced detachment of a significant number of trophozoites from the substrate (see above). In SEM analysis, drug-treated trophozoites that were still adhering appeared slightly condensed in shape. MTZ-treated trophozoites revealed flagella with an apparently reduced length (Fig. 4a, b). Orlistat-treated trophozoites showed a more undulated and blistered dorsal surface and a large number of flagella displayed terminal blebs which, in most cases, were single but rarely multiple blebs could be observed (Fig. 4c–d).

In addition, ultrastructural elements of the trophozoites were analysed by TEM of ultrathin sections through adherent and detached trophozoites. Drug-treated trophozoites showed general structural disintegration, like extracted cytoplasm and the presence of multilamellar bodies that were not specific for MTZ or orlistat but rather concentration-dependent (more affected cells with increasing MTZ or orlistat concentration; not shown). The cytoskeleton of the cilia and ventral disc was structurally not affected by either of the treatment, even if trophozoites showed already significant extraction of the cytoplasm (not shown). Quantitative changes to the overall shape and size of the median body could not be reliably analysed but the arrangement of microtubules within the median body seemed to be unaffected based on the approximately 20 trophozoite profiles per sample in which the median body was hit in the section. Differences in the Romanowsky staining of flagella and median body were therefore not due to a loss of the microtubular structures but may be related to other modifications induced by orlistat treatment, e.g. membrane permeabilization, extraction of cytosolic components or molecular modifications, which impair the staining.

### Orlistat has a Giardiacidal Effect *in vitro*

**Figure 5:**
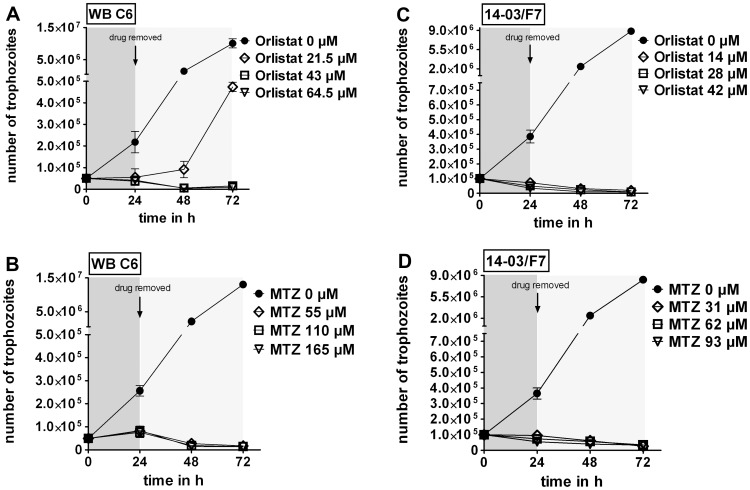
Killing of *G. duodenalis* trophozoites by orlistat. Giardiacidal effects of orlistat or MTZ were determined for drug concentrations indicated using *G. duodenalis* strains WB-C6 and 14-03/F7. WB-C6 trophozoites (5×10^4^) and 14-03/F7 trophozoites (1×10^5^) were treated with orlistat and MTZ, respectively, for 24 h and, after drug removal, cultured for another 48 h. Trophozoite numbers were determined every 24 h. (A) Orlistat was used at 5×(21.5 µM), 10×(43 µM), and 15×(64.5 µM) of the IC50_24h_ value for WB-C6. (B) Similarly, MTZ was used at 5×(55 µM), 10×(110 µM), and 15×(165 µM) of the IC50_24h_ concentration for WB-C6. (C) Orlistat was used at 5×(14 µM), 10×(28 µM), and 15×(42 µM) of the IC50_24h_ value for 14-03/F7. (D) Similarly, MTZ was used at 5×(31 µM), 10×(62 µM), and 15×(93 µM) of the IC50_24h_ concentration for 14-03/F7. Data are expressed as mean ± SD of parasite numbers determined in triplicate cultures. One of three experiments with similar results is shown (note the gap on the y-intercept).

To evaluate giardiacidal versus giardiastatic effects of orlistat *G. duodenalis* trophozoites were incubated for 24 h in the presence of 5×, 10×, and 15×the IC50_24h_ concentrations (i.e., 21.5, 43 and 64.5 µM orlistat for strain WB-C6 and 14, 28 and 42 µM orlistat for strain 14-03/F7). MTZ has been shown before to be giardiacidal [11] and was used as a control at equivalent multiples of IC50_24h_ doses (55 µM, 110 µM and 165 µM MTZ for WB-C6 and 31, 62 and 93 µM for strain 14-03/F7). As expected from our previous data, after one day of exposure both drugs caused total growth inhibition at all concentrations tested. The parasite numbers corresponded approximately to the inoculated trophozoite number or were even less (Fig. 5). Note that trophozoite counts included all cells that could be identified as trophozoites, irrespective of viability.

Further cultivation in growth medium in the absence of the drugs revealed that orlistat at concentrations higher than 43 µM (10×IC50_24h_) was sufficient to kill trophozoites of strain WB-C6, as no further parasite growth was detectable within 48 h (Fig. 5a). The 24 h-treatment with 5×IC50_24h_ orlistat did not kill all trophozoites, since during the recovery time of 48 h the trophozoites gained back their replication capability (Fig. 5a). In contrast, orlistat treatment at a concentration of 5×IC50_24h_ (14 µM) killed all trophozoites of isolate 14-03/F7 indicating strain or genotype-specific differences in orlistat sensitivity (Fig. 5b). The treatment of both *G. duodenalis* strains, WB-C6 and 14-03/F7, with MTZ at the 5×IC50_24h_ concentration was sufficient to kill all trophozoites (Fig. 5c, 5d).

### Independent Inhibition of *G. duodenalis* Growth by Orlistat and MTZ

**Figure 6:**
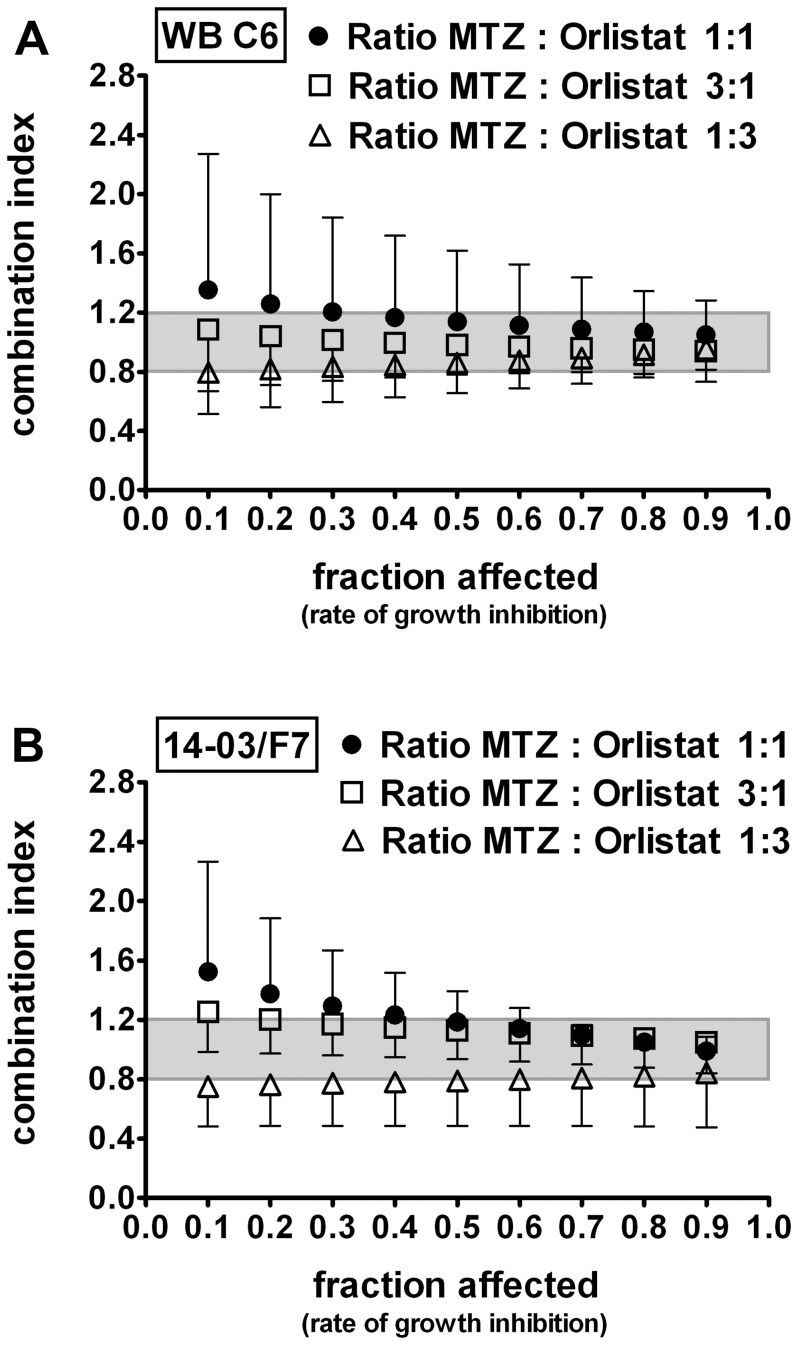
No interaction effect of MTZ and orlistat in combination on the growth of *G. duodenalis* trophozoites. Trophozoites of *G. duodenalis*, (A) WB-C6 (n = 4) and (B) 14-03/F7 (n = 3), were incubated in the presence of various concentrations of MTZ (0.33–81 µM) or orlistat (0.33–27 µM) alone or in combination. Combination indices (CIs) of MTZ and orlistat were calculated for given drug ratios (MTZ:orlistat) of 1∶1 (filled circle), 3∶1 (open squares), or 1∶3 (open triangle). Results are plotted against the fraction of parasites affected (normalized growth inhibition). Data are presented as mean ± SD of independent experiments (each performed in triplicates). CI values between 0.8–1.2 (shaded area) indicate no interaction of the drugs regarding growth inhibition (i.e. drugs act independently).

Next, the combined effect of orlistat and MTZ on *G. duodenalis* growth was tested. Parasite cultures of WB-C6 and 14-03/F7 were treated with the drugs individually over a range of concentrations (see methods) and in combination at different molar ratios (3∶1, 1∶1, or 1∶3, respectively). The combination index (CI), an indicative measure of synergistic, antagonistic or summation effects as defined by Chou and Talaley [36], was determined and plotted against the normalized effect ( = fraction affected) of the drug combinations on *G. duodenalis* growth (Fig. 6). We considered only CI of fraction affected values >0.5 since lower values are less relevant for clinical applications [37]. For both *G. duodenalis* strains tested the means of the calculated CI values (n ≥3) at fraction affected values >0.5 ranged between 0.8 and 1.2, indicative of no interaction effects of the drug combination orlistat and MTZ (Fig. 6a, 6b).

## Discussion

In the present study we show that the approved anti-obesity drug orlistat inhibits *G. duodenalis* growth *in vitro*. Based on the calculated molar IC50_24h_ values, orlistat was a significantly more potent growth inhibitor than MTZ, one of the standard drugs currently in use to treat giardiasis patients.

Treatment of giardiasis is still a challenge and alternative therapeutic options are necessary since a substantial proportion of patients (up to 20%) is refractory to MTZ treatment [3]. Reasons for treatment failures likely include parasite factors as differences of *in vitro* drug sensitivity between laboratory strains and field isolates and between different human isolates have been reported [4], [7], [8], [13]. However, the underlying mechanisms of resistance are not yet fully understood and it is currently not clear if and how *in vitro* generated data on drug sensitivity reflect the *in vivo* situation in patients. Moreover, comparison of reported drug sensitivity data from *in vitro* studies appears critical in view of methodological differences. For example, using a 24 h assay we determined four to five times higher IC50 values of MTZ for the *G. duodenalis* strain WB-C6 when compared with the values obtained by a similar technique with a prolonged incubation time of 72 h as previously reported [34]. Recently, the same group documented a shorter replication time of genotype AI strains (e.g., WB-C6) versus isolates of genotype AII, B or EIII [4]. In agreement with this data, the freshly isolated AII strain 14-03/F7 replicated slower in our study than the WB-C6 strain. It is currently not known whether different replication times *in vitro* are genetically determined and may therefore be correlated with similar differences *in vivo,* or whether a longer replication represents an indicator of stress under culture conditions. Interestingly, the ratio of the IC50_24h_ values of MTZ and orlistat were similar for the isolates WB-C6 and 14-03/F7 (2.6 vs. 2.2, respectively), indicating that the relative efficiency of the drugs was comparable on both *G. duodenalis* isolates. Future studies will be needed to systematically assess the clinical relevance of *in vitro* drug sensitivity values.

Our analysis of the combination effects suggests that the mode of action of MTZ and orlistat is independent. This was also supported by the specific structural changes as revealed by EM for the two drugs although the molecular reasons for these changes remain to be elucidated. With respect to clinical studies, it was important to analyse possible antagonistic interactions. The additive inhibitory effect of MTZ and orlistat on *G. duodenalis* provides a basis for clinical studies and suggests that a combined therapeutic application may be beneficial for patients. There is precedence from individual cases in which combinations of currently used antigiardial drugs have been successfully applied for the treatment of patients refractory to single medication [39]. It should be noted that *G. duodenalis* is able to rapidly develop resistance against drugs *in vitro* [11], [40]. Although a similar effect against orlistat cannot yet be excluded, the use of drugs in combination may be therapeutically advantageous in that the development of drug resistance is hampered. However, this will require comparative clinical studies.

Several criteria motivated us to consider orlistat as a promising candidate for the treatment of human giardiasis. The mode of action of orlistat in the treatment of obesity is linked to its covalent binding to the serine in the active side cleft of the conserved G-X-S-X-G motif present in pancreatic lipases [21], [41]. *G. duodenalis* has only a limited set of enzymes for de novo synthesis or remodelling of lipids and it is thought to be highly dependent on lipid supply from the host [19], [20], [42]. Mining of the currently available genomic data of the three fully sequenced *G. duodenalis* isolates (www.giardiadb.org) revealed several putative triglyceride lipases and phospholipases as potential targets of orlistat (data not shown and Vargas-Villarreal et al. [43]). These putative *G. duodenalis* lipases possess similar G-X-S-X-G motifs. Thus, orlistat may affect the parasite directly by inhibiting the parasite’s own lipid metabolizing enzymes and indirectly by limiting the lipid supply through the inhibition of host enzymes. A conserved motif similar to the G-X-S-X-G motif in lipases is present in fatty acid synthases (FAS) that are also potently inhibited by orlistat. Notably, inhibition of FAS by orlistat potently affects cancer cells and it is therefore currently considered as a leading compound for the development of new anti-cancer drugs [28], [44]. However, genome analysis revealed that *Giardia spp.* likely lack FAS and do not seem to be capable of *de novo* synthesis of fatty acids [19]. Although we cannot exclude off-target effects of orlistat in *G. duodenalis* we suggest that the demonstrated giardiacidal effect is due to the inhibition of parasite lipases. This statement is indirectly supported by our SEM analysis that revealed for orlistat a specific undulating and blistering effect on the trophozoite surface suggesting interference with the metabolism of lipids or other membrane components. However, the elucidation of the detailed mode of action of orlistat in *G. duodenalis* should be addressed in the future, as this was not the scope of the current study. Furthermore, findings that orlistat is highly effective against other pathogens including mycobacteria and protozoan parasites suggest a broad anti-microbial effect by yet unknown mechanisms [18], [27], [29], [45], [46].

Importantly, orlistat has a very poor intestinal absorption rate and remains highly effective in the intestinal lumen until it is excreted with the stool [26], [47]. Thus, in the case of giardiasis, this *in situ* bioavailability of orlistat should facilitate to sustain a sufficiently high drug concentration at the site of infection for the inhibition of parasite lipases. Our *in vitro* results revealed effective doses in the micromolar range for both orlistat and MTZ. A common treatment regimen with MTZ is 3×250 mg/d orally for five to seven days [3]. The drug is well absorbed and distributed, and in pharmacological studies reached at this dosing 20 µg/mL plasma peak levels [48] that are equivalent to 116 µM. This corresponds to ∼10× the IC50_24h_ that we determined for MTZ in this study. For orlistat, the recommended dose for obese patients is 120 mg ( = 0.24 mmol) three times a day. Assuming a net amount of fluid turnover in the human gut of approximately 10 L per day a rough estimation suggests that the drug may well reach a concentration of 72.6 µM on average in the intestine, which would be approximately 17 times higher than the IC50_24h_ value determined *in vitro* for *G. duodenalis* strain WB-C6. Of note, doses as high as 400 mg of orlistat have been administered in some clinical studies [26]. Thus, in analogy to MTZ, clinically evaluated dosage regimens for orlistat may be appropriate to reach giardiacidal level *in vivo*.

Orlistat is generally well tolerated but it should be noted that prolonged use can cause side effects [23]–[26], [47], [49] and importantly, can interfere with the absorption of concomitantly orally administered lipophilic drugs [25]. For example, the case of a HIV patient was recently presented who showed reduced control of HIV infection under antiretroviral therapy in co-medication with orlistat presumably due to reduced uptake of Efavirenz, a lipophilic component of the anti-viral therapy [50]. Although treatment with orlistat may thus be contraindicated in certain patients, we still believe that this is not a prohibitive argument against clinical studies regarding its benefit in MTZ-refractory *G. duodenalis* infections.

In conclusion, orlistat not only efficiently killed *G. duodenalis* trophozoites *in vitro* but was also more effective than MTZ. The demonstrated ‘no interaction’ effect of the drugs in combination may be beneficial in the treatment of patients suffering from giardiasis, i.e., reducing the extent of severe side effects caused by MTZ and the risk of drug resistance, while improving efficacy. Our data call for controlled clinical studies to evaluate the usefulness of orlistat in the treatment of giardiasis in humans and/or animals. This is the first report showing efficacy of orlistat against a microaerophilic protozoan pathogen. It may also have activity against other gastrointestinal parasites.
